# Recent Advances in Electrochemical Biosensors for the Detection of Foodborne Pathogens: Current Perspective and Challenges

**DOI:** 10.3390/foods12142795

**Published:** 2023-07-23

**Authors:** Bo Wang, Hang Wang, Xubin Lu, Xiangfeng Zheng, Zhenquan Yang

**Affiliations:** 1 College of Food Science and Engineering, Yangzhou University, Yangzhou 225009, China; wb@yzu.edu.cn (B.W.); zxf@yzu.edu.cn (X.Z.); 2 College of Veterinary Medicine, Yangzhou University, Yangzhou 225009, China; dx120180109@yzu.edu.cn; 3 College of Animal Science and Technology, Yangzhou University, Yangzhou 225009, China; lxb@yzu.edu.cn

**Keywords:** electrochemical biosensors, foodborne pathogens, food, environment, detection

## Abstract

Foodborne pathogens cause many diseases and significantly impact human health and the economy. Foodborne pathogens mainly include *Salmonella* spp., *Escherichia coli*, *Staphylococcus aureus, Shigella* spp., *Campylobacter* spp. and *Listeria monocytogenes*, which are present in agricultural products, dairy products, animal-derived foods and the environment. Various pathogens in many different types of food and water can cause potentially life-threatening diseases and develop resistance to various types of antibiotics. The harm of foodborne pathogens is increasing, necessitating effective and efficient methods for early monitoring and detection. Traditional methods, such as real-time polymerase chain reaction (RT-PCR), enzyme-linked immunosorbent assay (ELISA) and culture plate, are time-consuming, labour-intensive and expensive and cannot satisfy the demands of rapid food testing. Therefore, new fast detection methods are urgently needed. Electrochemical biosensors provide consumer-friendly methods to quickly detect foodborne pathogens in food and the environment and achieve extensive accuracy and reproducible results. In this paper, by focusing on various mechanisms of electrochemical transducers, we present a comprehensive overview of electrochemical biosensors for the detection of foodborne pathogens. Furthermore, the review introduces the hazards of foodborne pathogens, risk analysis methods and measures of control. Finally, the review also emphasizes the recent research progress and solutions regarding the use of electrochemical biosensors to detect foodborne pathogens in food and the environment, evaluates limitations and challenges experienced during the development of biosensors to detect foodborne pathogens and discusses future possibilities.

## 1. Introduction

Foodborne pathogens are pathogenic bacteria that can cause food poisoning or use food as a transmission medium [1]. Pathogenic bacteria directly or indirectly contaminate food and water sources, and oral infection in humans can lead to the occurrence of intestinal infectious diseases, food poisoning and the prevalence of infectious diseases in livestock and poultry [1]. *Salmonella* spp., *Escherichia coli*, *Staphylococcus aureus*, *Shigella* spp., *Campylobacter* spp. and *Listeria monocytogenes* are the major bacterial agents that cause foodborne infections [2]. As foodborne pathogens harm people’s health, people pay special attention to food safety and food biosecurity. On the one hand, foodborne pathogens in livestock, poultry and aquatic animals are treated or prevented by antibiotics, but some antibiotics are resistant to pathogenic bacteria; as a result, these antibiotics are not effective against pathogenic bacteria [3]. To protect customers from crippling and sometimes lethal instances of pathogen outbreaks, the safety of foods from farm to fork across the supply chain continuum must be guaranteed. The method of hazard analysis critical control points (HACCPs) is one preventive strategy that can be used to ensure safety; however, its full potential will not be reached unless the necessary supporting tools are created [4]. Therefore, a rapid, sensitive and accurate detection method combined with HACCPs must be established to improve the safety of foods.

The presence of preliminaries in ready-to-eat (RTE) foods is a serious problem because these products usually have not received any further treatment before consumption. In fact, outbreaks of foodborne pathogens originate from undercooked or processed RTE meats, dairy products, fruits and vegetables [5,6,7]. Agricultural products (vegetables and fruits), animal-derived foods (meat, milk and eggs) and the environment (water and soil) are the most important reservoirs for many foodborne pathogens [8,9,10,11,12]. Therefore, fruits, vegetables, seafood, meat, eggs and milk products may carry *Salmonella*, *Staphylococcus aureus*, *Campylobacter*, *Listeria*, *Shigella* or *Escherichia coli* O157:H7 organisms. The traditional detection methods commonly used for foodborne pathogens include polymerase chain reaction (PCR) [13,14,15], enzyme-linked immunosorbent assay (ELISA) [16,17,18] and culture plate [19]. However, traditional detection techniques are limited by disadvantages, such as large time costs, low efficiency and complex equipment. The test paper method exhibits several advantages, as it is efficient, portable and convenient to operate; thus, multiple foodborne pathogens in food and the environment can be quantitatively detected by this method [20]. Biosensor detection technology exhibits several advantages, including strong selectivity, high accuracy and short detection time, and has attracted widespread attention and been applied to detect foodborne pathogens [21,22,23,24,25]. Compared with traditional detection technology, advanced methods (test strips and biosensors) offer technological innovation and can efficiently, quickly and conveniently detect foodborne pathogens in food and the environment [26].

Electrochemical biosensors detect foodborne pathogens based on potentiometry, conductometry and impedimetry [27]. Due to their advantages, including rapid processes, high sensitivity, high specificity, low cost, portability, miniaturization and point-of-care detection, electrochemical biosensors have been widely used in the fields of food, biology and life sciences [28,29]. Electrochemical biosensors provide a rapid, efficient and alternative method for detecting foodborne pathogens to ensure the safety of RTE foods and can be used as stand-alone devices for on-site monitoring. Nanomaterials (NMs) employed in the fabrication and nanobiosensors include metallic nanoparticles, carbon nanotubes (CNTs), organic nanoparticles, metal oxide nanoparticles and silica nanoparticles [30]. Furthermore, these nanomaterials can act as transduction elements, thereby improving the sensitivity and detection limit of the electrochemical biosensor method [31,32]. Therefore, the selection of a highly specific bioreceptor in combination with a nanomaterial is essential for electrochemical biosensor development, which can quickly and efficiently detect foodborne pathogens [33].

This review attempts to provide a comprehensive overview of the detection of foodborne pathogens through rapid, sensitive and accurate electrochemical biosensor methods for food and environmental research. In addition, this review introduces the principle of electrochemical biosensors, focuses on the hazards, risk analysis and control of foodborne pathogens, discusses the recent progress and limitations of electrochemical biosensors in foodborne pathogen detection and proposes some solutions and future challenges.

## 2. Principle of Electrochemical Biosensors

**Figure 1:**
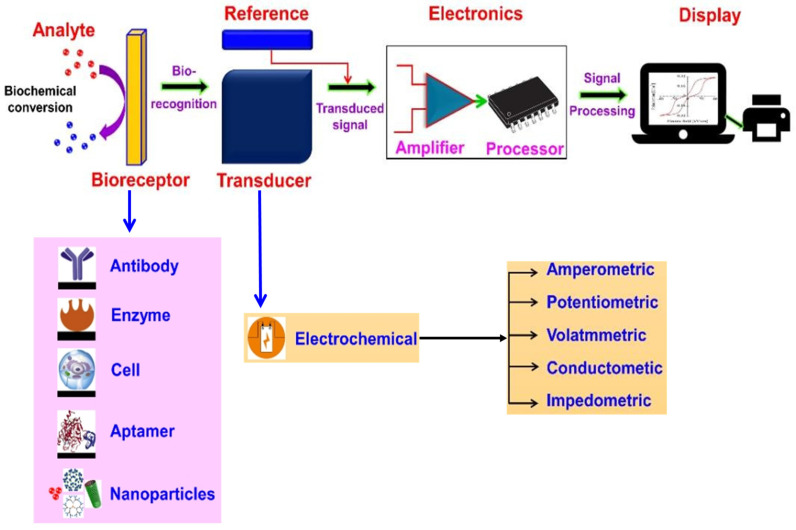
Schematic diagram of a typical electrochemical biosensor consisting of a bioreceptor, transducer, electronic system (amplifier and processor) and display (PC or printer) for the detection of foodborne pathogens. Adapted with permission from Naresh and Lee [32]. Copyright 2021, MDPI.

A typical electrochemical biosensor consists of an analyte (e.g., *Salmonella*, *Staphylococcus aureus*, *Campylobacter* and *Listeria*), bioreceptor (e.g., antibodies, enzymes, cells, aptamers and nanoparticles), electrochemical transducer, electronics and display [32,34]. The most extensively studied and applied class of biosensors, electrochemical biosensors, depend on the electrochemical nature of the analyte and the transducer for their operation [35]. The electrochemical biosensor is based on the principle that a bioreceptor and analyte interact electrochemically on the transducer surface, resulting in detectable electrochemical signals; this signal is measured in terms of voltage, current, impedance and capacitance, allowing for the quantitative or qualitative analysis of the analyte [36]. Figure 1 depicts the electrochemical biosensor’s working principle.

**Figure 2:**
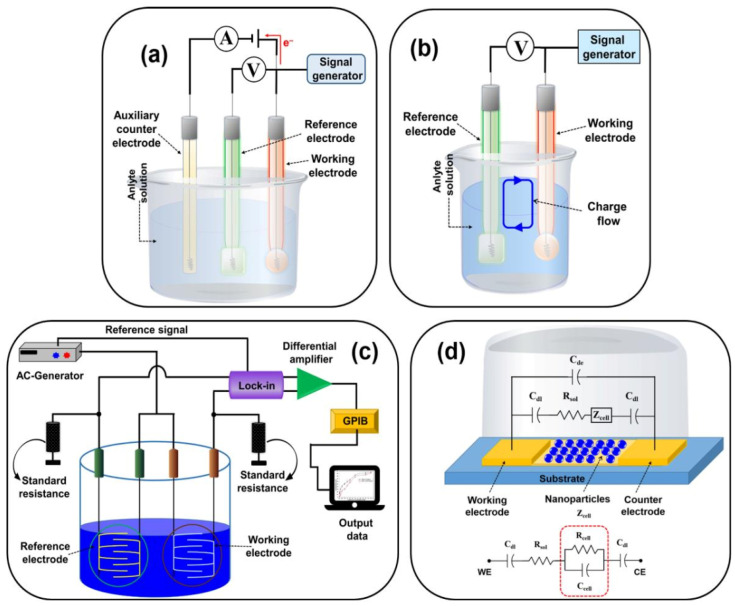
Schematic diagram of (**a**) amperometric/voltametric; (**b**) potentiometric; (**c**) conductometric biosensors; and (**d**) equivalent circuit of the impedimetric biosensor (C_dl_ = double-layer capacitance of the electrodes; R_sol_ = resistance of the solution; C_de_ = capacitance of the electrode; Z_cell_ = impedance introduced by the bound nanoparticles; R_cell_ and C_cell_ = the resistance and capacitance in parallel, respectively). Adapted with permission from Naresh and Lee [32]. Copyright 2021, MDPI.

The combination of various bioreceptors (antibodies, DNA, enzymes, microbes or cells) and electrochemical converters (current, potential, voltage, conductance and impedance) can constitute a variety of electrochemical biosensors. Electrochemical biosensors are divided into amperometric, potentiometric, voltammetric, conductometric and impedimetric biosensors based on the transduction principle [37]. Figure 2 shows schematic designs for the following different types of biosensors: (a) amperometric/voltammetric, (b) potentiometric, (c) conductometric and (d) impedimetric biosensors. Compared with other biosensors, electrochemical biosensors exhibit several advantages, including high sensitivity, good selectivity, fast response, small sample dosage and easy-to-achieve multicomponent measurement [38]. At present, electrochemical biosensor technology has been widely used in the detection of foodborne pathogens.

## 3. Foodborne Pathogens: Hazards, Risk Analysis and Control

Foodborne illness is a major cause of morbidity and continues to pose a serious danger to public health worldwide. Foodborne illnesses are most frequently caused by bacteria, which exhibit a range of sizes, varieties and characteristics. Foodborne illness starts with the production of breeding animals, vegetables and fruits during processing; it is then transported to the supermarket or farmer’s market and is finally passed to consumers. Therefore, based on the needs of consumers in production and processing, it is necessary to design an effective and safe food safety management system to control and reduce the harm and risks caused by foodborne bacteria. This review mainly introduces the hazards, risk analysis methods and measures used to control *Salmonella* spp., *Escherichia coli*, *Staphylococcus aureus, Shigella* spp., *Campylobacter* spp. and *Listeria monocytogenes*.

### 3.1. Salmonella spp.

Theobald Smith isolated *Salmonella* bacteria from pig intestines infected with classical swine fever in 1885 [39]. *Salmonella* is a flagellated Gram-negative, non-spore-forming bacillus and facultative anaerobe that thrives at temperatures from 35 to 37 °C [40]. *Salmonella* has a complex antigen structure, which can generally be divided into somatic antigen (O), flagella antigen (H) and surface antigen (Vi) [41]. This bacterium is well known as a foodborne pathogen because most infections are acquired through food. The bacteria cause salmonellosis, and the main symptoms include nausea, vomiting, abdominal pain, headache, chills and diarrhoea [42]. The people who are the most likely to be infected with *Salmonella* are infants or children under 5 years of age, elderly individuals and immune-damaged people [39]. Salmonellosis can be acquired from the ingestion of food and water contaminated with *Salmonella* or exposure to an environment contaminated with faeces containing *Salmonella* [43]. The consumption of undercooked food from infected animals in poultry products, other meats, raw milk, dairy products made from raw milk, RTE foods (such as fruits and vegetables contaminated with faeces of infected animals) or water contaminated with the faeces of infected people or animals could all be sources of contamination [43,44]. *Salmonella* infection is a common outbreak of diseases worldwide, including in European and American countries [45,46]. Therefore, the study of *Salmonella* has always been a hot topic. To prevent an outbreak of salmonellosis, we can take some preventive measures, including introducing sanitary environments at farms, treating faeces in a no-risk manner and treating feed and water [47]. During the breeding process, some antibiotics and vaccines can be used to inhibit the growth of *Salmonella*, but attention must be focused on the amount of antibiotics, the dosage period and elimination law [48,49]. Based on the growth temperature of *Salmonella*, pathogenic bacteria can be killed at high temperature [40]. To control *Salmonella*, people can use high-temperature cooking methods when preparing animal products, such as meat, milk and eggs.

### 3.2. Escherichia coli

*Escherichia coli* (*E. coli*) is a Gram-negative, facultative anaerobic rod that inhabits the intestinal tract of animals and humans from birth [50]. *E. coli* is a member of the natural microbial community of the animal and human gut. It produces useful vitamins and competes with and inhibits the growth of pathogenic bacteria that may be present or consumed with food and water, among other beneficial functions in the body [40]. Many of these *E. coli* strains are not pathogenic, and only a small part causes various diseases of animals and humans under certain conditions [51]. According to serological classification, *E. coli* strains can be divided into somatic antigen (O), flagellar antigen (H) and capsule antigen (K) [52]. Based on the mechanism by which the gastrointestinal pathogenic *E. coli* causes illnesses, it is divided into the following major foodborne diarrhoeagenic *E. coli* pathotypes: Shiga toxin-producing *E. coli*/enterohemorrhagic *E. coli* (STEC/EHEC), enteropathogenic *E. coli* (EPEC), enteroinvasive *E. coli* (EIEC), enterotoxigenic *E. coli* (ETEC) and enteroaggregative *E. coli* (EAEC) [53]. Pathogenic *E. coli* strains can cause intestinal gastroenteritis, urinary tract infections, meningitis infections and blood infections [52]. The sickness caused by the bacterium *E. coli*, which typically lives in the lower intestines of most warm-blooded mammals, is known as “colibacillosis.” It is mainly caused by infections, such as specific bacterial wool antigen and pathogenic toxins. *E. coli* has been utilized as a sign of faecal contamination for almost a century since it is one of the predominant enteric species in human faeces, in addition to anaerobic bacteria [40]. The concept of indicators is based on the premise that the presence of *E. coli* in food or water is proof that it has been faeces-contaminated and may also be evidence of the presence of pathogens. Although the use of *E. coli* as a faecal indicator has been criticized for being unreliable because it can be found in environmental sources, it is nevertheless used as an indicator of cleanliness throughout the world because no adequate replacement has been suggested. In recent years, pathogenic *E. coli* has caused many foodborne outbreaks in industrialized countries via the faecal–oral route because it is consumed in contaminated meat, vegetables, fruits and water [54]. O157:H7 and some of the other pathogenic *E. coli* families have been well documented for transmitting secondary infections through animal or person-to-person contact [55]. *E. coli* has been exposed to antibiotics for a long time in humans and animal intestines; as a result, *E. coli* is resistant to many antibiotics (β-lactams, quinolones, aminoglycosides, tetracyclines, sulphonamides and phenicols) [56]. From a One Health perspective, antimicrobial resistance in *E. coli* is a problem of the utmost concern because it affects both the human and animal sectors. Considering the causes of pathogenic *E. coli* and drug resistance, some measures can be taken to control the bacteria, such as sterilizing milk and juice through the Pakistani method, cooking meat and effectively washing RTE foods.

### 3.3. Staphylococcus aureus

**Figure 3:**
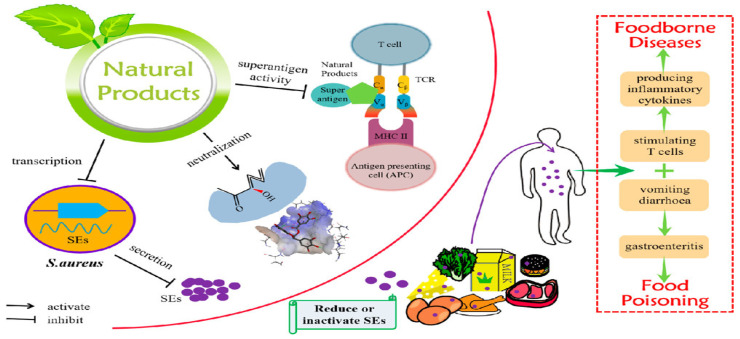
Mechanism by which natural products prevent foodborne diseases induced by SEs. Adapted with permission from Liu et al. [64]. Copyright 2022, American Chemical Society.

The genus Staphylococcus contains more than 30 species, of which *Staphylococcus aureus* (*S. aureus*) has the greatest effect on human health [57]. *S. aureus* is a common Gram-positive bacterium with a diameter of approximately 1 μm [58]. The temperature and pH range for the growth of *S. aureus* are 7–49 °C and 4–9, respectively, and the best growth temperature and pH are 30–37 °C and 7, respectively [44]. *S. aureus* is a serious bacterial pathogen that can lead to a wide range of illnesses, including food poisoning, toxic shock syndrome, wound infections and skin infections [59]. *S. aureus* is a common dweller (commensal) of the skin, nares, respiratory tracts and genitalia of both humans and animals [59]. However, as an opportunistic pathogen, it can cause invasive and deadly infections in a variety of organs. A significant amount of extracellular proteins and toxins are produced by *S. aureus*. Given that many *S. aureus* strains produce enterotoxins, the growth and spread of *S. aureus* in foods pose a potential risk to consumer health [60]. The most significant toxins are known as staphylococcal enterotoxins (SEs) and SE-like toxins (SEls), and these toxins have the following factors in common: they are structurally identical proteolytic enzymes that are resistant to heat, are superantigenic and exert emetic effects [61,62]. In addition, drug-resistant *S. aureus* strains have become one of the most common pathogens recovered from hospital-associated (nosocomial) infections, which is of particular public health concern [63]. Due to the medicinal resistance and heat resistance of these enterotoxins, the treatment and control of *S. aureus* remains a challenge. Therefore, the main goal should be to stop *S. aureus* from growing and contaminating food. According to the growth conditions of *S. aureus*, deep cooking can effectively prevent the harm caused by *S. aureus*. Additionally, a number of natural products can be employed to effectively lower the toxicity of SEs and the prevalence of foodborne diseases; these products can also serve as food antibacterial agents in place of antibiotics and chemical preservatives [64]. In Figure 3, Liu et al. [64] presented information on the toxicity of SEs, the types of food that are contaminated by SEs and the sources and methods by which SEs can contaminate food. This information will help to manage and lower the rate by which SEs contaminate food.

### 3.4. Shigella spp.

*Shigella* are pathogens that originate in the Escherichia genus but are commonly categorized as a different genus [65]. *Shigella* spp. are Gram-negative bacteria that cause the intestinal infection known as shigellosis [66]. Shigella may grow at pH levels of 6 to 8 and in a wide range of temperatures (from 10 to 48 °C) [67]. It is possible to isolate *Shigella* spp. from a variety of food sources, and it causes several outbreaks and sporadic cases of foodborne diseases worldwide. Typically, moist items touched with bare hands, such as salads, uncooked veggies, fruits, shellfish and water, are linked to shigellosis [68]. The most common symptoms of shigellosis are diarrhoea, fever, nausea, vomiting, gastrointestinal bloating and constipation [69]. Shigella species and EIEC both produce diarrhoeal illnesses using the same invasive mechanism [70]. *Shigella* spp. can cause many people to develop and even show high mortality, which seriously endangers public health [71,72,73]. Shigella infection therapy with antibiotics is crucial for lowering the disease’s prevalence and fatality rates [74]. Ciprofloxacin is recommended by the World Health Organization (WHO) as a first-line treatment for shigellosis, and second-line treatments include azithromycin, ceftriaxone or pivmecillinam [75]. However, many antibiotics have caused the strains of Shigella to produce multidrug resistance, including β-lactams, fluoroquinolones, macrolides, tetracyclines and phenicols, thereby limiting the effects of their antibiotic resistance to severe infection [76,77,78,79,80,81,82]. Some preventive measures for foodborne shigellosis include the removal of faeces, ensuring safe drinking water, developing good personal hygiene habits, avoiding cross-infection of RTE foods and using appropriate water–chloride-washed vegetables for salad and refrigerated food. WHO, a global institution that has extensively focused on this subject, has emphasized the significance of creating an effective vaccination against Shigella. Due to the multidrug resistance of *Shigella* spp., scientific researchers are developing vaccines to produce corresponding antibodies by activating the body’s immune system, thereby effectively controlling these pathogenic strains of Shigella [63,83]. It is believed that these vaccines developed for Shigella can pass clinical trials in the future and reduce mortality.

### 3.5. Campylobacter spp.

*Campylobacter* (*C.*) spp., Gram-negative bacteria, are responsible for human acute gastroenteritis (campylobacteriosis) worldwide, with most cases being caused by *C. jejuni* and *C. coli* [84,85]. The optimal pH and temperature range of *Campylobacter* growth are 6.5–7.5 and 37–42 °C, respectively. Compared to Salmonella or pathogenic *E. coli*, the number of cases caused by *Campylobacter* is much greater [86]. Human infection mainly manifests as symptoms of acute enteritis, such as diarrhoea, discomfort, fever, abdominal pain and blood in stools. Current Campylobacteriosis outbreaks have been linked to meat, raw milk, fruits and vegetables [87,88]. The main source of *Campylobacter* transmission in humans is poultry, specifically broiler chickens, which contain the highest concentrations of this bacteria [89,90]. One of the primary public health policies in the EU aimed at preventing campylobacteriosis is to manage this disease in poultry and poultry meat [91]. This suggests that controlling *Campylobacter* in chickens at the farm level can reduce the danger of human exposure to this virus and significantly improve food safety. It would be very interesting to see how biosecurity measures could reduce environmental exposure [92]. In slaughterhouses, waste can be disinfected when chickens are slaughtered, and the packaging carton can be disinfected to prevent transmission to humans by transportation. To control the spread of *Campylobacter* on farms, either the prevalence of infected broiler flocks must be reduced or the amount of the pathogen in the broilers’ intestines must be reduced before slaughter [93]. Although *Campylobacter* exhibits resistance to some antibiotics, antibiotic use remains an effective measure to control *Campylobacter* spp. [94]. In addition, reuterin is a broad-spectrum antimicrobial system produced by specific strains of *Lactobacillus reuteri* during the anaerobic metabolism of glycerol, which can effectively inhibit the potential of *Campylobacter* spp. [95]. Furthermore, antibiotics are anticipated to be replaced by plant-, animal-, bacterial- and marine-derived antimicrobials to suppress *Campylobacter* spp. [96]. The comprehensive approach (longitudinally integrated safety assurance model, LISA) across the farm–slaughterhouse–processing–retail–consumer continuum is the suggested method for preventing and controlling *Campylobacter* along the poultry meat chain [93].

### 3.6. Listeria monocytogenes

*Listeria monocytogenes* (*Lm*) is a Gram-positive, rod-shaped and psychotropic bacterium that causes listeriosis, a very uncommon but potentially fatal gastrointestinal illness [97]. The temperature and pH range for the growth of *Lm* are 0–45 °C and 4.1–9.6, respectively, and the optimum growth temperature is 30–35 °C [53]. The bacterium *Lm* has been found in a variety of environments and foods, including water, soil, sewage, silage, pasteurized milk, various fruits and vegetables and several meat products. With a high foodborne proportion of up to 99%, the consumption of infected food products is the primary method by which listeriosis is transmitted to humans [98]. *Lm* bacteria usually lead to intestinal infection, causing patients to show symptoms such as fever, muscle soreness, nausea and vomiting. It can also invade the nervous system and circulatory system, causing severe meningitis and sepsis [99]. The outbreak of listeriosis seriously endangers human health and causes economic losses. Therefore, the European Union has developed food safety criteria (Commission Regulation (EC) 2073/2005) for *Lm* in RTE foods. Although some antibiotics show resistance to *Lm*, the use of antibiotics remains one of the most common methods for treating *Lm*. Amoxicillin or ampicillin, frequently in conjunction with gentamicin, is the mainstay therapy for severe infections caused by *Lm*, and cotrimoxazole, fluoroquinolones, rifampicin and linezolid are alternatives to aminopenicillins [100]. To reduce *Lm* infection, regular disinfection must be performed in breeding environments, including the pollution-free treatment of faeces [101]. In addition, some natural or synthetic compounds can inhibit the formation of *Lm* biofilms, which is also a novel strategy [102]. Utilizing natural antimicrobial agents, which can serve as a viable replacement for synthetic preservatives for the production of organic food products, is among the alluring and efficient ways to limit the growth of *Lm* in food items [103].

In summary, the use of antibiotics or natural antibacterial agents can inhibit foodborne pathogens. To prevent the infection of foodborne pathogens, the consumption of RTE foods should be minimized, and the products should be disinfected and sealed during the entire food production chain. In addition, it is important to develop effective, fast and sensitive analysis methods to quickly identify foodborne pathogens in food and the environment.

## 4. Electrochemical Biosensors for the Detection of Foodborne Pathogens in Food and the Environment

This review focuses on different bioreceptors combined with electrochemical transducers to measure six types of foodborne pathogens in food and the environment. Enzymes, DNAs/RNAs, aptamers and antibodies are frequently used in bioreceptor applications [104]. In addition, numerous studies have employed nanomaterial-based biosensors for the detection of foodborne pathogens [105]. Based on different bioreceptors, we summarized the development of electrochemical biosensors for the detection of foodborne pathogens in the past ten years (2013–2023), aiming to provide the latest trends in this research field. Table 1 summarizes some published electrochemical biosensor methods for the detection of foodborne pathogens in food and the environment.

### 4.1. DNA-Based Electrochemical Biosensors

Genosensors are DNA biosensors that utilize hybridization processes to identify certain nucleic acids in bacterial cells and detect the analyte [159]. Over the past ten years, DNA probe diagnostic testing has emerged as a technology with great potential for pathogen identification and analysis in food samples. Since there is no chance of detecting antigens or antibodies, as is typically performed in physiological samples, direct detection in the genetic fragment is achievable by nanosensors with probes containing nucleic acids, which are strongly advised against ingestion [160]. To increase sensitivity and specificity, DNA-coated nanomaterials are frequently used in probes, which can frequently detect bacterial RNA without amplifying it [161].

**Figure 4:**
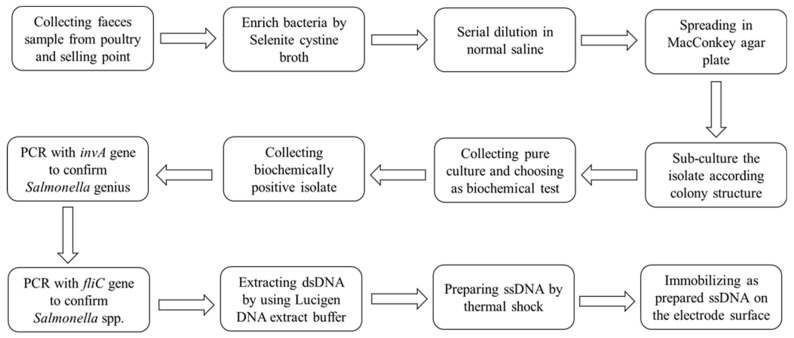
Detailed work flow for *S*. Typhi sample collection and identification. Adapted with permission from Bacchu et al. [114]. Copyright 2022, Elsevier.

Bacchu et al. [114] developed a DNA-based biosensor for detecting *Salmonella* Typhi (*S. typhi*) in blood, poultry faeces, eggs and milk by using DNA-immobilized modified SPE. The biosensor was created by immobilizing an amine-labelled single-stranded DNA (ssDNA) probe specific to *S*. Typhi on the surface of the P-Cys@AuNP-modified SPE. They introduced a process to quickly and efficiently extract ssDNA, and the whole process lasted approximately 2 h (Figure 4). This biosensor uses the DPV technique to determine *S*. Typhi complementary-target DNA sequences. The linear response in the actual sample was 1.8–1.8 × 10^5^ CFU/mL, and the LOD value was 1 CFU/mL. The excellent recoveries in the spiked sample were 96.54–103.47%, indicating that the biosensor could detect *S*. Typhi in food and clinical samples. The combination of ssDNA probes and nanomaterials provides the selectivity, stability, reproducibility and regeneration of electrochemical biosensors, which should be applied to detect other foodborne pathogens.

As DNA-based biosensors have advanced in the detection of food pathogens, Pangajam et al. [134] developed a novel electrochemical sensor based on a CDs/ZnO nanoroad/PANI nanoassembly for the detection of *E. coli* O157:H7 in water samples. It was discovered that the exceptional electrical conductivity of CD/ZnO/PANI increased the sensitivity for the detection of *E. coli*. The successful detection of *E. coli* O157:H7 in water samples was achieved using the developed electrochemical biosensor, which also showed good selectivity and had a detection limit of 1.3 × 10^−18^ M. A rapid and sensitive analysis for *LM* in ham samples was achieved by Li et al. [153] through a phosphatase (ALP)-mediated magnetic relaxation DNA biosensor. This magnetic biosensor demonstrated great sensitivity for *LM* detection with a linear range from 2 × 10^2^ to 2 × 10^7^ CFU/mL and an LOD of 10^2^ CFU/mL without requiring any DNA amplification steps.

### 4.2. Electrochemical Immunosensors

Antibody–antigen biosensors, commonly referred to as immunosensors, are frequently used analytical instruments for the detection of foodborne pathogens in food [162]. The immobilization of a particular anti-pathogen antibody on the surface of a transducer serves as the basis for this biosensor’s operation. When an antigen is coupled to the antibody, an immunochemical reaction occurs that serves as the signal for biosensor detection [163]. Recently, electrochemical immunosensors have been widely used in the detection of different foodborne pathogens in foods.

**Figure 5:**
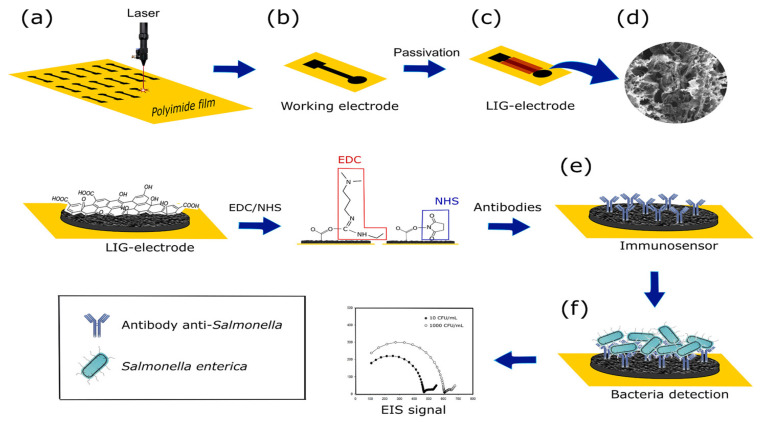
Fabrication, biofunctionalization and sensing scheme of the LIG immunosensor. The fabrication and biofunctionalization steps included: (**a**) LIG processing onto a polyimide (Kapton) sheet to create the working electrode; (**b**) working electrode; (**c**) passivation of the working electrode with lacquer; (**d**) SEM image showing the LIG surface; (**e**) biofunctionalization with *Salmonella* antibodies immobilized on the working electrode via carbodiimide cross-linking chemistry; and (**f**) *Salmonella* binding to the electrode and the resultant Nyquist plot generated during electrochemical sensing. Adapted with permission from Soares et al. [110]. Copyright 2020, American Chemical Society.

A label-free biosensor was developed by Soares et al. [110] for the detection of Salmonella enterica in chicken broth. To identify *S. enterica* Typhimurium by carbodiimide cross-linking, a bare laser-induced graphene (LIG) electrode was functionalized with polyclonal antibodies on its surface (Figure 5). This study used the EIS method to determine *S. enterica* Typhimurium in chicken broth. The analysis time was 22 min, the linear range of the method was 10^1^–10^5^ CFU/mL and the detection limit was 13 CFU/mL. In this research, a low-cost, sensitive and selective electrochemical immunosensor method was developed to determine foodborne pathogens in food, which provides an important contribution to food safety.

Mo et al. [136] described a novel sensitive and quantitative sandwich electrochemical immunosensor technique for the detection of E. coli O157:H7 using immune gold@platinum nanoparticles (Au@Pt), neutral red (NR), rGO nanocomposite and regenerative leucoemeraldine-based PANI/AuNP-modified SPCE. Although the SPCE’s disposable nature was replaced by the potential for reuse, its batch-manufacturing benefits were still present. Based on electrochemical detection of *E. coli* O157:H7, the linear range of the method was 8.9 × 10^3^–8.9 × 10^9^ CFU/mL, with an LOD of 2.84 × 10^3^ CFU/mL. To further evaluate the quantitative detection capacity of biosensors, this study conducted a spiked recovery experiment on milk and pork samples. The recovery of spiked milk and pork samples exceeded 78.6%, showing the good precision and reliability of the immunosensor. Similarly, Lu et al. [151] developed an enzyme-labelled amperometric immunosensor for the detection of *Lm* by immobilizing an HRP-labelled antibody against *Lm* onto the surface of novel MWCNT fibres. Milk samples were spiked with *Lm* bacteria, and qualitative results detecting contamination were presented. The linear range of the method was from 10^2^ to 10^5^ CFU/mL (𝑅^2^ = 0.993), and the LOD was 1.07 × 10^2^ CFU/mL. The potential use of the immunosensor for the quick detection of *LM* was further demonstrated by its good storage stability and reproducibility (RSD < 6.5%).

### 4.3. Electrochemical Aptasensors

Short DNA or RNA molecules known as aptamers exhibit a high affinity and selectivity when binding to their target molecules, which can include drugs, proteins, toxins, sugar, antibiotics and bacteria [164]. Compared to RNA aptamers, DNA aptamers are more stable and are widely used in electrochemical aptasensors to detect foodborne pathogens in food and the environment. Compared to the manufacture of antibodies, the synthesis of aptamers exhibits numerous advantages because it is quick, inexpensive, does not involve animal products and does not generate batch-to-batch fluctuations. DNA aptamers typically have a high affinity for their target, are resistant to high temperatures, are stable over time and are simple to modify by chemical groups for immobilization or labelling purposes.

**Figure 6:**
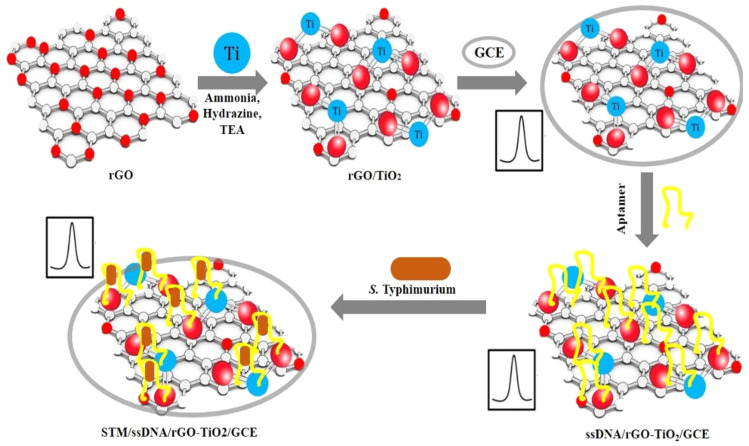
A schematic diagram of the stepwise fabrication of rGO-TiO_2_ electrodes and electrochemical detection of bacteria. Adapted with permission from Muniandy et al. [117]. Copyright 2019, Elsevier.

*S. typhimurium* was detected by Muniandy et al. [117] in chicken meat samples using rGO-TiO_2_ nanocomposite-based electrochemical aptasensors (Figure 6). The bacterial cells are linked to the DNA aptamer that has been adsorbed on the rGO-TiO_2_ surface, creating a physical barrier that prevents electron transmission. This study used the DPV method to identify *S. typhimurium*. The optimized aptasensor demonstrated good selectivity for Salmonella bacteria, great sensitivity, a wide detection range (10^1^–10^8^ CFU/mL) and an LOD of 10 CFU/mL. Wang et al. [133] developed an electrochemical aptasensor using a coaxial capillary with magnetic nanoparticles, urease catalysis and a PCB electrode for the rapid and sensitive detection of *E. coli* O157:H7 in milk samples. This aptasensor obtained a good recovery (>99.7%) and precision (RSD, 1.4%–4.3%), and the LOD was 10 CFU/mL. In another study, Abbaspour et al. [144] introduced a sensitive and highly selective dual-aptamer-based sandwich immunosensor for the detection of *S. aureus*. Due to its short detection time, high sensitivity and low cost, this proposed aptasensor offers the potential for practical applications in the detection of foodborne pathogens.

### 4.4. CRISPR/Cas-Based Electrochemical Biosensor

As a method for genome editing, CRISPR is employed to treat numerous diseases [27]. However, with advancements in research, CRISPR combined with electrochemical biosensors has been utilized for the detection of *Salmonella*, *E. coli* O157:H7, *S. aureus* and *Lm* in food [107,129,137,154]. Recently, CRISPR/Cas-based methods for the detection of *Salmonella*, *E. coli* O157:H7, *S. aureus* and *Lm* were created, although these methods remain in their very early stages and need to be further developed. To our knowledge, the Cas9, Cas12a, Cas12b, Cas13a and Cas13b proteins are mainly used in the detection of foodborne pathogens [165,166].

**Figure 7:**
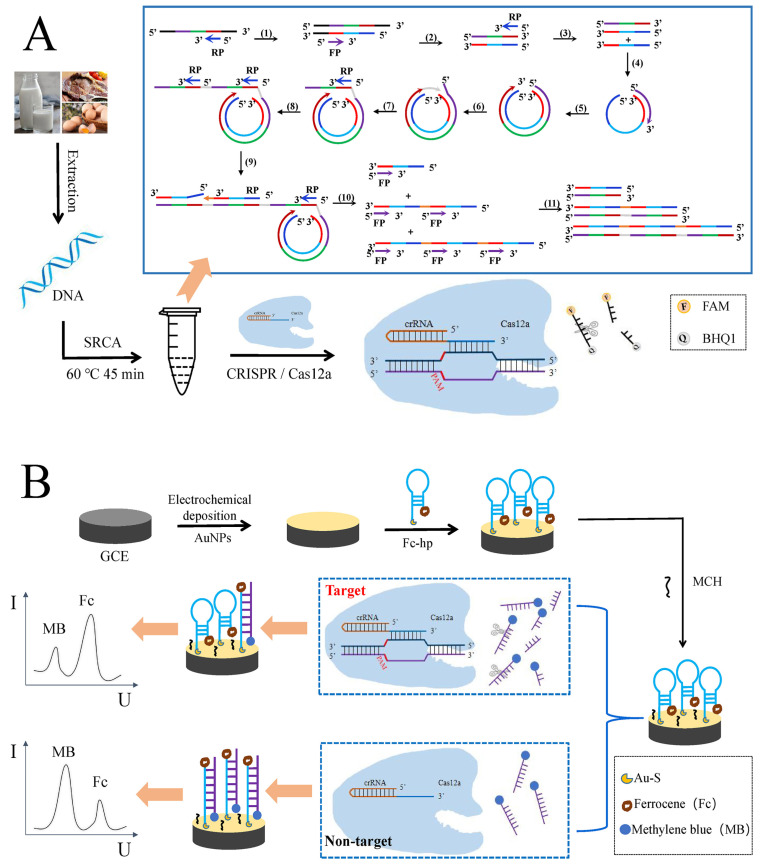
(**A**) Schematic diagram of the SRCA-CRISPR/Cas12a assay; (**B**) schematic diagram of the biosensor for the detection of Salmonella. Adapted with permission from Zheng et al. [107]. Copyright 2023, Elsevier.

Zheng et al. [107] first reported a ratiometric electrochemical biosensor based on the SRCA-CRISPR/Cas12a system for the detection of *Salmonella* (Figure 7). This strategy can effectively use the target’s particular Cas12a-crRNA binding and eliminate nonspecific amplification. The specificity and sensitivity of traditional SRCA responses are greatly improved by the combination of SRCA and CRISPR/Cas12a. The linear range of the method was 5.8 fg/μL–5.8 ng/μL, and the LOD was 2.08 fg/μL. For the detection of actual samples (chicken and pork), this biosensor exhibited good sensitivity, precision and specificity, and the detection results of this biosensor were consistent with real-time fluorescent quantitative PCR (RT-qPCR). Overall, the biosensor offers a useful platform for the extremely accurate and sensitive detection of *Salmonella* in food, with the potential to also monitor other foodborne pathogens. In another study, Chen et al. [129] developed an electrochemical biosensor based on CRISPR/Cas12a combined with immuno-RCA for the detection of *E. coli* O157:H7. The developed biosensor presented a broad linear range from 10 to 10^7^ CFU/mL, with an LOD of 10 CFU/mL. Compared to traditional electrochemical DNA sensors, the CRISPR Cas system based on electrochemical DNA sensors is higher in terms of sensitivity and precision [167]. It also exhibits complementarity between CRISPR and electrochemical-sensing technology.

Recently, Huang et al. [137] introduced a novel electrochemical biosensor based on SRCA combined with the CRISPR/Cas12a system for the accurate detection of *S. aureus*. In the presence of *S. aureus*, the target DNA double strands obtained by SRCA can be specifically identified with the Cas12a/crRNA complex. The accidental cleavage characteristic of Cas12a is activated by this combination, which amplifies the reporting signal. The sensitivity and specificity of the method is significantly enhanced by this step. The linear range of the method was 3.9 × 10^1^–3.9 × 10^7^ CFU/mL, and the LOD was 3 CFU/mL. For the detection of actual milk samples, the recoveries were 98.8–117.1%, which justified the good accuracy of this biosensor. It provides a highly specific and ultrasensitive detection platform for foodborne pathogens. Similarly, Li et al. [154] developed an ultrasensitive CRISPR/Cas12a-based electrochemical biosensor (E-CRISPR) combined with recombinase-assisted amplification (RAA) for the detection of *Lm*. The results indicated that this method has a good linearity (9.4 × 10^0^–9.4 × 10^7^ CFU/mL) and sensitivity (LOD, 9.4 × 10^2^ CFU/g). Compared to previous Cas12a-based signal amplification strategies, the RAA-based E-CRISPR platform not only took full advantage of the specific RNA recognition ability of Cas12a to achieve high specificity, but also converted the target recognition activity into a detectable electrochemical signal to improve the sensitivity.

## 5. Conclusions and Outlooks

Foodborne pathogenic microorganisms in food and the environment are an issue that warrants attention and are related to human health and safety. To ensure the food safety of consumers, it is very important to develop rapid and efficient biosensor technology to effectively determine food pathogens in food and the environment. Therefore, we provided a comprehensive review of electrochemical biosensors for the detection of food pathogens in food and the environment (2013–2023). Compared with traditional detection methods (RT-PCR, ELISA and culture plates), electrochemical biosensors exhibit several advantages, as the technique exhibits high sensitivity, achieves real-time detection and is selective, rapid and inexpensive. This review focused on the detection principle behind electrochemical biosensors, the hazards of food pathogens, risk analysis and control measures and recent progress. Various combinations of materials and methods have been used to develop different bioreceptor-based sensors for the detection of six kinds of food pathogens. As shown in Table 1, this review of bioreceptors, detection methods, assay strategies and material types analysed the current advanced electrochemical biosensors for the detection of food pathogens in different sample matrices. With the development of nanomaterials, good nanomaterials are fixed to the electrode to improve the sensitivity, selectivity and stability of electrochemical biosensors. Due to some of the shortcomings of bioreceptors, there are certain limitations of electrochemical biosensors, such as low stability for antibodies, restriction to DNA targets for nucleic acids and sensitivity to nuclease for aptamers. However, the combination of CRISPR technology and electrochemical DNA sensors successfully improved the sensitivity and precision. Biosensor-based devices have become an important part of the equipment used in laboratories to detect biological responses. In spite of having created a variety of biosensors for detecting foodborne pathogens, it is still difficult to design biosensors for the reliable and effective determination of microorganisms in real food samples. In practical applications, some electrochemical biosensors can only detect single food samples, which may result from the complexity of the animal-derived food matrix. In addition, available research reports indicate that these electrochemical biosensors still have a problem with simultaneously detecting the number of food pathogens. Overall, a biosensor’s essential characteristics include sensitivity, specificity, stability, detection time, sample processing, size and the capacity to function under a variety of settings without the need for specialized training. Although electrochemical biosensors must be further developed to solve these problems, cooperation between scientific researchers and enterprises can pave the way for the development of a desirable, portable product. The development of food safety biosensors will significantly improve people’s quality of life and health.

## Figures and Tables

**Table 1:** An overview of some reported electrochemical biosensors used for the detection of foodborne pathogens in food and the environment.

Target Pathogen	Bioreceptor	Detection Method	Assay Strategy	Material Type	LOD	Linear Range	Matrix	Ref.
*Salmonella* spp.	DNA probe	SWV–CV–EIS	SPIA-based biosensors	AuNPs/GCE	68 CFU/mL	6.8 × 10^1^–6.8 × 10^8^ CFU/mL	Animal meat	[106]
*Salmonella* spp.	DNA probe	SWV	SRCA-CRISPR/Cas12a signal amplification strategy	AuNPs/GCE	2.08 fg/μL	5.8 fg/μL–5.8 ng/μL	Chicken and pork	[107]
*Salmonella* spp.	Aptamer	DPV	Aptasensor	Gold nanoparticles	200 CFU/mL	2 × 10^2^–2 × 10^6^ CFU/mL	Milk	[108]
*Salmonella* spp.	Aptamer	CV–EIS–DPV	Aptasensor	rGO-AuNPs	200 CFU/mL	6 × 10^2^–6 × 10^7^ CFU/mL	Pork and beef	[109]
*Salmonella* spp.	Antibody	EIS	Immunosensors	Multilayer graphene	13 CFU/mL	10^1^–10^5^ CFU/mL	Chicken broth	[110]
*Salmonella* spp.	Antibody	DPV	Immunosensor	CoFe-MOFs-graphene	1.2 × 10^2^ CFU/mL	2.4 × 10^2^–2.4 × 10^8^ CFU/mL	Milk	[111]
*S. enteritidis*	Bacteriophages as new molecular probes	EIS	Phage-based biosensor	GDE-AuNPs-Cys-Phage SEP37	1 CFU/mL	2 × 10^2^–2 × 10^5^ CFU/mL	Chicken breast meat	[112]
*S. pullorum* and *S. gallinarum*	Antibody	CV	Immunosensor	SPCE	16.1 CFU/mL	10^1^–10^9^ CFU/mL	Chicken and eggs	[113]
*S. typhi*	DNA probe	DPV	DNA biosensor	SPE/P-Cys@AuNPs	1 CFU/mL	1.8–1.8 × 10^5^ CFU/mL	Blood, poultry faeces, eggs and milk	[114]
*S. typhimurium*	Magnetosome-anti-Salmonella antibody complex	EIS	Magnetosome-based biosensors	SPCE	10^1^ CFU/mL	10^1^–10^7^ CFU/mL	Water and milk	[115]
*S. typhimurium*	Antibody	CV–EIS	Immunosensor	AuNPs/PAMAM-MWCNT-Chi/GCE	5.0 × 10^2^ CFU/mL	1.0 × 10^3^–1.0 × 10^7^ CFU/mL	Milk	[116]
*S. typhimurium*	Aptamer	DPV	Aptasensor	rGO-TiO_2_ nanocomposite	10^1^ CFU/mL	10^1^–10^8^ CFU/mL	Chicken meat	[117]
*S. typhimurium*	DNA probe	SWV–CV–EIS	SRCA-based ratiometric electrochemical biosensor	SH-β-CD/AuNPs/GCE	15.8 fg/μL	30 fg/μL–30 ng/μL	Animal meat, eggs and dairy products	[118]
*S. typhimurium*	Antibody	SWV	Immunosensor	SPCE	4 CFU/mL	4–36 CFU/mL	Milk	[119]
*S. typhimurium*	Aptamer	CV–EIS	Aptasensor	AuNPs/GCE	1 CFU/mL	6.5 × 10^2^–6.5 × 10^8^ CFU/mL	Eggs	[120]
*E. coli*	Engineered phage	DPV	Bacteriophage-based biosensors	SWCNT-SPE	1 CFU/mL	1–10^4^ CFU/mL	Spinach leaves	[121]
*E. coli*	L-cysteine	CV	Amino functionalized iron nanoparticles-based biosensors	L-Cyst-Fe_3_O_4_ NPs	10 CFU/mL	10^1^–10^5^ CFU/mL	Tap water	[122]
*E. coli*	PNA probe	Conductometry	DNA biosensor	AuNPs	10^2^ CFU/mL	10^3^–10^8^ CFU/mL	Water	[123]
*E. coli*	Aptamer-primer probe	CV–DPV	RCA coupled DNAzyme amplification-based biosensor	Au	8 CFU/mL	9.4–9.4 × 10^5^ CFU/mL	Milk	[124]
*E. coli*	Antibody	CV–EIS	Immunosensor	AuSPEs	30 CFU/mL	10^1^–10^8^ CFU/mL	Drinking water	[125]
*E. coli*	Aptamer	DPV	Aptasensor	Au	80 CFU/mL	5.0 × 10^2^–5.0 × 10^7^ CFU/mL	Licorice extract	[126]
*E. coli*	Antibody	EIS	MOF based biosensor	Ab/Cu_3_(BTC)_2_-PANI/ITO	2 CFU/mL	2.0–2 × 10^8^ CFU/mL	Lake water	[127]
*E. coli*	Aptamer-NanoZyme	CV	Aptamer-NanoZyme based biosensor	AuNPs	10 CFU/mL	10^1^–10^9^ CFU/mL	Apple juice	[128]
*E. coli* O157:H7	DNA probe	DPV	CRISPR/Cas12a- and immuno-RCA-based biosensors	Au	10 CFU/mL	10^1^–10^7^ CFU/mL	Milk	[129]
*E. coli* O157:H7	Aptamer	CV–EIS–DPV	Aptasensor	Au	10 CFU/mL	10^1^–10^6^ CFU/mL	Milk	[130]
*E. coli* O157:H7	Dual-DNA probe	CV–EIS–DPV	Dual-DW biosensor	Au	30 aM	10^−7^–10^−1^ nM	Peach juice and milk	[131]
*E. coli* O157:H7	Dual-DNA probe	SWV	Dual-DW biosensor	Polyaniline nanopillar array	10 CFU/mL	10^1^–10^5^ CFU/mL	Milk	[132]
*E. coli* O157:H7	Aptamer	Impedimetry	Aptasensor	MNPs-AuNPs	10 CFU/mL	10^1^–10^5^ CFU/mL	Milk	[133]
*E. coli* O157:H7	DNA probe	DPV	DNA hybridization biosensors	CD/ZnO/PANI	1.3 × 10^−18^ M	1.3 × 10^−18^–5.2 × 10^−12^ M	Water	[134]
*E. coli* O157:H7	Aptamer	EIS	Aptasensor	3D-IDEA	2.9 × 10^2^ CFU/mL	10^1^–10^5^ CFU/mL	Drinking water	[135]
*E. coli* O157:H7	Antibody	CV	Immunosensor	SPCE-PANI-AuNPs	2.84 × 10^3^ CFU/mL	8.9 × 10^3^–8.9 × 10^9^ CFU/mL	Milk and pork	[136]
*S. aureus*	DNA probe	SWV	SRCA-CRISPR/Cas12a -based E-DNA biosensor	AuNPs/GCE	3 CFU/mL	3.9 × 10^1^–3.9 × 10^7^ CFU/mL	Milk	[137]
*S. aureus*	DNA probe	EIS	Aptasensor	rGO-AuNPs	10 CFU/mL	10–10^6^ CFU/mL	Fish and water	[138]
*S. aureus*	DNA probe	DPV	SDA reaction and triple-helix molecular switch based biosensor	Au	8 CFU/mL	30–3 × 10^8^ CFU/mL	Lake water, tap water and honey	[139]
*S. aureus*	IgG	EIS	Label-free ECL biosensor	Carboxyl graphene/porcin IgG/GCE	3.1 × 10^2^ CFU/mL	10^3^–10^9^ CFU/mL	Milk, lake water, human saliva and human urine	[140]
*S. aureus*	Aptamer	CV–EIS	Aptasensor	AuNPs/CNPs/CNFs	1 CFU/mL	1.2 × 10^1^–1.2 × 10^8^ CFU/mL	Human serum	[141]
*S. aureus*	DNA probe	DPV	DNA biosensor	MWCNT-Chi-Bi	3.17×10^−14^ M	3.87 × 10^−14^–1.22 × 10^−15^ M	Beef	[142]
*S. aureus*	Antibody	CV–DPV	Paper-based immunosensor	SWCNT	13 CFU/mL	10–10^7^ CFU/mL	Milk	[143]
*S. aureus*	Aptamer	DPV	Aptasensor	AgNPs	1 CFU/mL	10–10^6^ CFU/mL	Tap and river water	[144]
*S. aureus*	Dual-DNA probe	DPV	DNA walker and DNA nanoflowers based biosensor	Au	9 CFU/mL	60–6 × 10^7^ CFU/mL	Lake water, tap water and honey	[145]
*Shigella flexneri*	DNA probe	CV–EIS–DPV	DNA biosensor	ITO/P-Mel/PGA/DSS	10 cells/mL	80–8 × 10^10^ Cells/mL	Meat, milk, bread, tape water and salad	[146]
*Shigella dysenteriae*	Aptamer	EIS	Aptasensor	GCE/AuNPs	1 CFU/mL	10^1^–10^6^ CFU/mL	Water and milk	[147]
*Campylobacter* spp.	DNA probe	CV–SWV	Genosensor	COP/Au	90 pM	1–25 nM	Raw poultry meat	[148]
*L. monocytogenes*	Antibody	CV–EIS	Immunosensor	SAM/Au	10^2^ CFU/mL	10^3^–10^6^ CFU/mL	Milk	[149]
*L. monocytogenes*	DNA probe	CV	DNA biosensor	CNF/AuNPs	82 fg/6 µL	0–0.234 ng/6 μL	Milk	[150]
*L. monocytogenes*	Antibody	CV	Immunosensor	MWCNT fibres	1.07 × 10^2^ CFU/mL	10^2^–10^5^ CFU/mL	Milk	[151]
*L. monocytogenes*	Antibody	EIS	Immunosensor	IDE/MBs-AuNPs	30 CFU/mL	3.0 × 10^1^–3.0 × 10^4^ CFU/mL	Lettuce	[152]
*L. monocytogenes*	DNA probe	CV	DNA biosensor	MNPs	10^2^ CFU/mL	2 × 10^2^–2 × 10^7^ CFU/mL	Ham	[153]
*L. monocytogenes*	DNA probe	SWV	CRISPR/Cas12a-based biosensor	Au	9.4 × 10^2^ CFU/g	9.4 × 10^0^–9.4 × 10^7^ CFU/mL	Flammulina velutipes	[154]
*L. monocytogenes*	Ferric ammonium citrate and esculin	Amperometry	SCC based biosensor	Pt	-	10^2^–10^8^ CFU/mL	Milk	[155]
*L. monocytogenes*	Antibody	EIS	Immunosensor	IDE Au	5.5 CFU/mL	1 × 10^2^–2.2 × 10^3^ CFU/mL	Milk	[156]
*L. monocytogenes*	DNA probe	DPV	DNA biosensor	ssDNA/RGO/AuNPs/CILE	3.17 × 10^–14^ M	10^–13^–10^–6^ M	Fish meat	[157]
*L. monocytogenes*	Polyclonal antibody	EIS	Impedance biosensor	MNP(MAb)-Lm-AuNPs (urease-PAb)/SPIE	1.6 × 10^3^ CFU/mL	1.9 × 10^3^–1.9 × 10^6^ CFU/mL	Lettuce	[158]

Abbreviations: limit of detection, LOD; differential pulse voltammetry, DPV; poly cysteine, P-Cys; colony-forming units, CFU; electrochemical impedance spectroscopy, EIS; screen-printed carbon electrode, SPCE; single primer isothermal amplification, SPIA; square wave voltammetry, SWV; cyclic voltammetry, CV; glassy carbon electrode, GCE; saltatory rolling circle amplification, SRCA; clustered regularly interspaced short palindromic repeats-associated, CRISPR–Cas; reduced graphene oxide, rGO; thiol-modified β-cyclodextrin, SH-β-CD; immuno-rolling circle amplification, immuno-RCA; single-wall carbon nanotube-modified screen-printed electrode, SWCNT-SPE; L-cysteine, L-Cyst; Nanoparticles, NPs; peptide nucleic acid, PNA; dual-DNA walker, dual-DW; gold screen printed electrodes, AuSPEs; magnetic nanoparticles, MNPs; carbon dot, CD; polymerizing aniline, PANI; metal–organic frameworks, MOFs; indium–tin oxide, ITO; three-dimensional interdigitated electrode array, 3D-IDEA; polyaniline, PANI; strand displacement amplification, SDA; electrochemiluminescent, ECL; immunoglobulin G, IgG; carbon nanoparticles, CNPs; cellulose nanofibers nanocomposite, CNFs; multiwalled carbon nanotubes–chitosan–bismuth, MWCNT-Chi-Bi; poly melamine, P-Mel; poly-glutamic acid, PGA; disuccinimidyl suberate, DSS; cyclo Olefin Polymer, COP; self-assembled monolayers, SAM; interdigitated electrode, IDE; magnetic beads, MBs; magnetic nanoparticles, MNPs; somatic cell count, SCC; interdigitated electrode, IDE; carbon ionic liquid electrode, CILE; monoclonal antibody, MAb; polyclonal antibody, PAbs; screen-printed interdigitated electrode, SPIE.

## Data Availability

Not applicable.
